# Supplementary material for: Enhanced Parameter Estimation with Periodically Driven Quantum Probe
Source: Entropy (Basel). 2021 Oct 12;23(10):1333. doi: 10.3390/e23101333 (PMC8534368; doi:10.3390/e23101333)
Supplement: Supplementary file 1 [file entropy-23-01333-s001.zip › entropy-1374220-supplementary.pdf]

Supplemental Material for "Enhanced Parameter Estimation with Periodically Driven Quantum Probe"

Peter A. Ivanov[1]
[1]*Department of Physics, St. Kliment Ohridski University of Sofia, James Bourchier 5 blvd, 1164 Sofia, Bulgaria*

DIAGONALIZATION OF THE EFFECTIVE HAMILTONIAN

Normal Modes

Here we provide the explicit diagonalization of the effective Hamiltonian. Up to terms of order of Φ^{-2} the effective time-independent Hamiltonian is

$$\hat{H}_{\rm eff} = \hat{H}_0 + \frac{1}{\Phi}[\hat{v}, \hat{v}^{\dagger}] - \frac{1}{2\Phi^2}\{[[\hat{H}_0, \hat{v}], \hat{v}^{\dagger}] + [[\hat{H}_0, \hat{v}^{\dagger}], \hat{v}] + O(\Phi^{-3}). \quad (1)$$

We find

$$\begin{aligned}\hat{H}_{\rm eff} &= \omega_x \hat{a}_x^{\dagger}\hat{a}_x + \omega_y \hat{a}_y^{\dagger}\hat{a}_y + \frac{\Delta}{2}\sigma_z - \frac{4g_x g_y}{\Phi}\sigma_z(\hat{a}_x^{\dagger}+\hat{a}_x)(\hat{a}_y^{\dagger}+\hat{a}_y) \\ &\quad -\frac{2g_x^2\Delta}{\Phi^2}\sigma_z(\hat{a}_x^{\dagger}+\hat{a}_x)^2 - \frac{2g_y^2\Delta}{\Phi^2}\sigma_z(\hat{a}_y^{\dagger}+\hat{a}_y)^2. \end{aligned} \quad (2)$$

The effective Hamiltonian is diagonal in the spin basis. We assume that the spin is initially prepared in the state $|\psi_{\rm spin}\rangle = |\uparrow\rangle$. Next, we introduce position and momentum operators for each of the bosonic modes,

$$\hat{\hat{x}} = \frac{1}{\sqrt{2\omega_x}}(\hat{a}_x^{\dagger}+\hat{a}_x), \quad \hat{\hat{p}}_x = i\sqrt{\frac{\omega_x}{2}}(\hat{a}_x^{\dagger}-\hat{a}_x), \quad (3)$$

$$\hat{\hat{y}} = \frac{1}{\sqrt{2\omega_y}}(\hat{a}_y^{\dagger}+\hat{a}_y), \quad \hat{\hat{p}}_y = i\sqrt{\frac{\omega_x}{2}}(\hat{a}_y^{\dagger}-\hat{a}_y), \quad (4)$$

such that the effective Hamiltonian becomes

$$\begin{aligned}\hat{H}_{\rm eff} &= \frac{1}{2}(\hat{\hat{p}}_x^2 + \hat{\hat{p}}_y^2) + \frac{\omega_x^2}{2}\left(1 - \frac{8g_x^2\Delta}{\omega_x\Phi^2}\right)\hat{\hat{x}}^2 \\ &\quad + \frac{\omega_y^2}{2}\left(1 - \frac{8g_y^2\Delta}{\omega_y\Phi^2}\right)\hat{\hat{y}}^2 - \frac{8g_x g_y}{\Phi}\sqrt{\omega_x\omega_y}\hat{\hat{x}}\hat{\hat{y}} \end{aligned} \quad (5)$$

Finally, we can rewrite (5) as $\hat{H}_{\rm eff} = \frac{1}{2}(\hat{\hat{p}}_x^2 + \hat{\hat{p}}_y^2) + \frac{1}{2}\sum_{kl} B_{kl}\hat{\hat{q}}_k\hat{\hat{q}}_l$ where $\hat{\hat{q}} = \{\hat{\hat{x}}, \hat{\hat{y}}\}$ and

$$B_{kl} = \begin{bmatrix} \omega_x^2\left(1 - \frac{8g_x^2\Omega}{\omega_x\Phi^2}\right) & -\frac{8g_x g_y}{\Phi}\sqrt{\omega_x\omega_y} \\ -\frac{8g_x g_y}{\Phi}\sqrt{\omega_x\omega_y} & \omega_y^2\left(1 - \frac{8g_y^2\Omega}{\omega_y\Phi^2}\right) \end{bmatrix}. \quad (6)$$

The matrix B_{kl} is real and symmetric with non-negative eigenvalues ν_α^2 ($\alpha = 1, 2$). The eigenvectors are defined by $\sum_k B_{nk}b_k^{(\alpha)} = \omega^2\nu_\alpha^2 b_n^{(\alpha)}$, where we assume for simplicity $\omega_x = \omega_y = \omega$. We find $\nu_1^2 = 1-\lambda^2+O(\Phi^{-2})$ and $\nu_2^2 = 1+\lambda^2+O(\Phi^{-2})$ where $\lambda = \sqrt{\frac{8g_x g_y}{\omega\Phi}}$ is the dimensionless coupling. The corresponding eigenvectors are $b^{(1)} = \{1/\sqrt{2}, 1/\sqrt{2}\}$ and $b^{(2)} = \{1/\sqrt{2}, -1/\sqrt{2}\}$. In terms of normal mode coordinates $\{\hat{Q}_\alpha, \hat{P}_\alpha\}$ related via $\hat{\hat{q}}_k = \sum_{\alpha=1}^{2} b_k^{(\alpha)}\hat{Q}_\alpha$ and $\hat{\hat{p}}_k = \sum_{\alpha=1}^{2} b_k^{(\alpha)}\hat{P}_\alpha$ the Hamiltonian becomes $\hat{H}_{\rm eff} = \frac{1}{2}\left(\hat{P}_1^2 + \omega^2\nu_1^2\hat{Q}_1^2\right) + \frac{1}{2}\left(\hat{P}_2^2 + \omega^2\nu_2^2\hat{Q}_2^2\right)$. Finally, we introduce a new set of two bosonic field operators by the relation

$$\hat{Q}_\alpha = \frac{1}{\sqrt{2\omega\nu_\alpha}}(\hat{d}_\alpha^{\dagger} + \hat{d}_\alpha), \quad \hat{P}_\alpha = i\sqrt{\frac{\omega\nu_\alpha}{2}}(\hat{d}_\alpha^{\dagger} - \hat{d}_\alpha), \quad (7)$$

which bring the effective Hamiltonian into the diagonal form

$$\hat{H}_{\text{eff}} = \omega \sum_{\alpha=1}^{2} \nu_\alpha \hat{d}_\alpha^\dagger \hat{d}_\alpha, \tag{8}$$

where we have omitted the constant terms. The two sets of bosonic operators $\{\hat{a}_x, \hat{a}_y\}$ and $\{\hat{d}_1, \hat{d}_2\}$ may be expressed in terms of one another as

$$\begin{aligned}
\hat{d}_1 &= \frac{1}{\sqrt{2}}\{\cosh(\theta_1)\hat{a}_x + \sinh(\theta_1)\hat{a}_x^\dagger\} + \frac{1}{\sqrt{2}}\{\cosh(\theta_1)\hat{a}_y \\
&\quad + \sinh(\theta_1)\hat{a}_y^\dagger\}, \\
\hat{d}_2 &= \frac{1}{\sqrt{2}}\{\cosh(\theta_2)\hat{a}_x + \sinh(\theta_2)\hat{a}_x^\dagger\} - \frac{1}{\sqrt{2}}\{\cosh(\theta_2)\hat{a}_y \\
&\quad + \sinh(\theta_2)\hat{a}_y^\dagger\},
\end{aligned} \tag{9}$$

where the mixing angle is given by $\theta_\alpha = \frac{1}{2}\ln(\nu_\alpha)$.

Time-evolution

Here we provide derivation of the Heisenberg equations of motion for the normal mode operators. We have

$$\frac{d\hat{Q}_\alpha}{dt} = \hat{P}_\alpha, \quad \frac{d\hat{P}_\alpha}{dt} = -\omega^2\nu_\alpha^2\hat{Q}_\alpha, \tag{10}$$

such that the time-evolution of the expectation values are $\langle\hat{Q}_1(t)\rangle = A\sin(\omega\nu_1 t) + B\cos(\omega\nu_1 t)$ and respectively $\langle\hat{Q}_2(t)\rangle = C\sin(\omega\nu_2 t) + D\cos(\omega\nu_2 t)$. For concreteness, we assume that the two bosonic modes are initially prepared in the state $|\psi(0)\rangle = \frac{1}{2}(|0_x\rangle + i\,|1_x\rangle)(|0_y\rangle + i\,|1_y\rangle)$, which gives

$$\langle\hat{Q}_1(t)\rangle = \frac{\sin(\omega\nu_1 t)}{\sqrt{\omega}\nu_1}, \quad \langle\hat{Q}_2(t)\rangle = 0. \tag{11}$$

Similarly, we find that

$$\begin{aligned}
\langle\hat{Q}_1^2(t)\rangle &= \frac{1}{\omega}\{1 + \frac{1}{\nu_1^2}\left(\frac{3}{2} - \nu_1^2\right)\sin^2(\omega\nu_1 t)\}, \\
\langle\hat{Q}_2^2(t)\rangle &= \frac{1}{\omega}\{1 + \frac{1}{\nu_2^2}\left(\frac{1}{2} - \nu_2^2\right)\sin^2(\omega\nu_2 t)\}.
\end{aligned} \tag{12}$$

STEADY-STATE

Bosonic Quadratures

Here we provide the detail information of the expectation values of the experimental observables in the steady-state regime. Consider the vector operator defined by $\hat{h} = \{\hat{a}_x, \hat{a}_x^\dagger, \hat{a}_y, \hat{a}_y^\dagger\}^{\text{T}}$. We find that the time-evolution of $\hat{h}$ obeys the following equation

$$\partial_\tau\langle\hat{h}\rangle = \hat{G}_0\langle\hat{h}\rangle + a, \tag{13}$$

where $\tau = \omega t$ and

$$\hat{G}_0 = \begin{bmatrix}
-i\left(1 - \frac{\lambda^2}{2}\epsilon\right) - \tilde{\gamma} & i\frac{\lambda^2}{2}\epsilon & i\frac{\lambda^2}{2} & i\frac{\lambda^2}{2} \\
-i\frac{\lambda^2}{2}\epsilon & i\left(1 - \frac{\lambda^2}{2}\epsilon\right) - \tilde{\gamma} & -i\frac{\lambda^2}{2} & -i\frac{\lambda^2}{2} \\
i\frac{\lambda^2}{2} & i\frac{\lambda^2}{2} & -i\left(1 - \frac{\lambda^2}{2}\epsilon\right) - \tilde{\gamma} & i\frac{\lambda^2}{2}\epsilon \\
-i\frac{\lambda^2}{2} & -i\frac{\lambda^2}{2} & -i\frac{\lambda^2}{2}\epsilon & i\left(1 - \frac{\lambda^2}{2}\epsilon\right) - \tilde{\gamma}
\end{bmatrix}, \tag{14}$$

with $\epsilon = \Delta/\Phi$, $\tilde{\gamma} = \gamma/\omega$ and $a = \{-i\tilde{f}/2, i\tilde{f}/2, 0, 0\}^{\mathrm{T}}$.

In the steady state regime where $\partial_\tau \langle \hat{h} \rangle = 0$ we obtain $\langle \hat{h} \rangle_{\mathrm{ss}} = -\hat{G}_0^{-1} a$. Using this, the position and momentum quadratures are given by

$$\langle \hat{x} \rangle_{\mathrm{ss}} = \frac{\tilde{f}(1+\tilde{\gamma}^2 - \epsilon\lambda^2)}{(1-\epsilon^2)(\lambda^2 + \lambda_{-\mathrm{c}}^2)(\lambda^2 - \lambda_{+\mathrm{c}}^2)},$$
$$\langle \hat{y} \rangle_{\mathrm{ss}} = \frac{\tilde{f}\lambda^2}{(1-\epsilon^2)(\lambda^2 + \lambda_{-\mathrm{c}}^2)(\lambda^2 - \lambda_{+\mathrm{c}}^2)} \tag{15}$$

and $\langle \hat{p}_x \rangle_{\mathrm{ss}} = \tilde{\gamma} \langle \hat{x} \rangle_{\mathrm{ss}}$, $\langle \hat{p}_y \rangle_{\mathrm{ss}} = \tilde{\gamma} \langle \hat{y} \rangle_{\mathrm{ss}}$ with $\lambda_{\pm,\mathrm{c}}^2 = (1+\tilde{\gamma}^2)(1 \pm \epsilon)^{-1}$. Note that up to first order of Φ^{-1} we have $\lambda_{+,\mathrm{c}}^2 = \lambda_{-,\mathrm{c}}^2 = \lambda_{\mathrm{c}}^2$ where $\lambda_{\mathrm{c}}^2 = 1 + \tilde{\gamma}^2$.

Covariance Matrix Elements

In order to evaluate the steady state covariance matrix elements we write the set of coupled differential equations for the following bosonic operators:

$$\partial_\tau \langle \hat{a}_x^2 \rangle = -2\{i\left(1 - \frac{\lambda^2}{2}\epsilon\right) + \tilde{\gamma}\}\langle \hat{a}_x^2 \rangle + i\lambda^2\epsilon\langle \hat{n}_x \rangle + i\frac{\lambda^2}{2}\epsilon$$
$$+ i\lambda^2(\langle \hat{a}_x \hat{a}_y^\dagger \rangle + \langle \hat{a}_x \hat{a}_y \rangle) - i\tilde{f}\langle \hat{a}_x \rangle,$$
$$\partial_\tau \langle \hat{n}_x \rangle = -2\tilde{\gamma}\langle \hat{n}_x \rangle + i\frac{\lambda^2}{2}\epsilon(\langle \hat{a}_x^{\dagger 2} \rangle - \langle \hat{a}_x^2 \rangle) + i\frac{\lambda^2}{2}(\langle \hat{a}_x^\dagger \hat{a}_y \rangle$$
$$+ \langle \hat{a}_x^\dagger \hat{a}_y^\dagger \rangle - \langle \hat{a}_x \hat{a}_y^\dagger \rangle - \langle \hat{a}_x \hat{a}_y \rangle) - i\frac{\tilde{f}}{2}(\langle \hat{a}_x^\dagger \rangle - \langle \hat{a}_x \rangle). \tag{16}$$

The set of equations for $\langle \hat{a}_y^2 \rangle$ and $\langle \hat{n}_y \rangle$ are identical in form to (16) by replacing $x \leftrightarrow y$. Note that we assume the force term displaces only the x bosonic mode. Finally, the set of equations for the correlations between the two bosonic modes are

$$\partial_\tau \langle \hat{a}_x \hat{a}_y \rangle = -2\{i\left(1 - \frac{\lambda^2}{2}\epsilon\right) + \tilde{\gamma}\}\langle \hat{a}_x \hat{a}_y \rangle + i\frac{\lambda^2}{2}\epsilon(\langle \hat{a}_x^\dagger \hat{a}_y \rangle$$
$$+ \langle \hat{a}_x \hat{a}_y^\dagger \rangle) + i\frac{\lambda^2}{2}(\langle \hat{n}_x \rangle + \langle \hat{n}_y \rangle + \langle \hat{a}_x^2 \rangle + \langle \hat{a}_y^2 \rangle)$$
$$+ i\frac{\lambda^2}{2} - i\frac{\tilde{f}}{2}\langle \hat{a}_y \rangle,$$
$$, \quad \partial_\tau \langle \hat{a}_x \hat{a}_y^\dagger \rangle = -2\tilde{\gamma}\langle \hat{a}_x \hat{a}_y^\dagger \rangle + i\frac{\lambda^2}{2}\epsilon(\langle \hat{a}_x^\dagger \hat{a}_y^\dagger \rangle - \langle \hat{a}_x \hat{a}_y \rangle)$$
$$+ i\frac{\lambda^2}{2}(\langle \hat{a}_y^{\dagger 2} \rangle - \langle \hat{a}_x^2 \rangle + \langle \hat{n}_y \rangle - \langle \hat{n}_x \rangle) - i\frac{\tilde{f}}{2}\langle \hat{a}_y^\dagger \rangle. \tag{17}$$

One can solve the system in the steady state by setting all time derivatives to zero. Then the symmetric covariance matrix elements are given by

$$V_{11} = \frac{2\lambda_{\mathrm{c}}^4 - \lambda^4}{2(\lambda_{\mathrm{c}}^4 - \lambda^4)}, \quad V_{22} = \frac{2\lambda_{\mathrm{c}}^4 + (\lambda_{\mathrm{c}}^2 - 3)\lambda^4}{2(\lambda_{\mathrm{c}}^4 - \lambda^4)},$$
$$V_{12} = \frac{\tilde{\gamma}\lambda^4}{2(\lambda_{\mathrm{c}}^4 - \lambda^4)}, \tag{18}$$

and $V_{11} = V_{33}$, $V_{22} = V_{44}$, $V_{12} = V_{34}$. The other elements are

$$V_{13} = \frac{\lambda_{\mathrm{c}}^2\lambda^2}{2(\lambda_{\mathrm{c}}^4 - \lambda^4)}, \quad V_{24} = \frac{\lambda^6 - \lambda_{\mathrm{c}}^2\lambda^2}{2(\lambda_{\mathrm{c}}^4 - \lambda^4)},$$
$$V_{14} = \frac{\tilde{\gamma}\lambda_{\mathrm{c}}^2\lambda^2}{2(\lambda_{\mathrm{c}}^4 - \lambda^4)}, \tag{19}$$

and $V_{23} = V_{14}$.
